# Supplementary material for: A Simple and Cost-Efficient Platform for a Novel Porcine Circovirus Type 2d (PCV2d) Vaccine Manufacturing
Source: Vaccines (Basel). 2023 Jan 12;11(1):169. doi: 10.3390/vaccines11010169 (PMC9865830; doi:10.3390/vaccines11010169)
Supplement: Supplementary file 1 [file vaccines-11-00169-s001.zip › vaccines-2085135-supplementary.pdf]

Supplementary materials

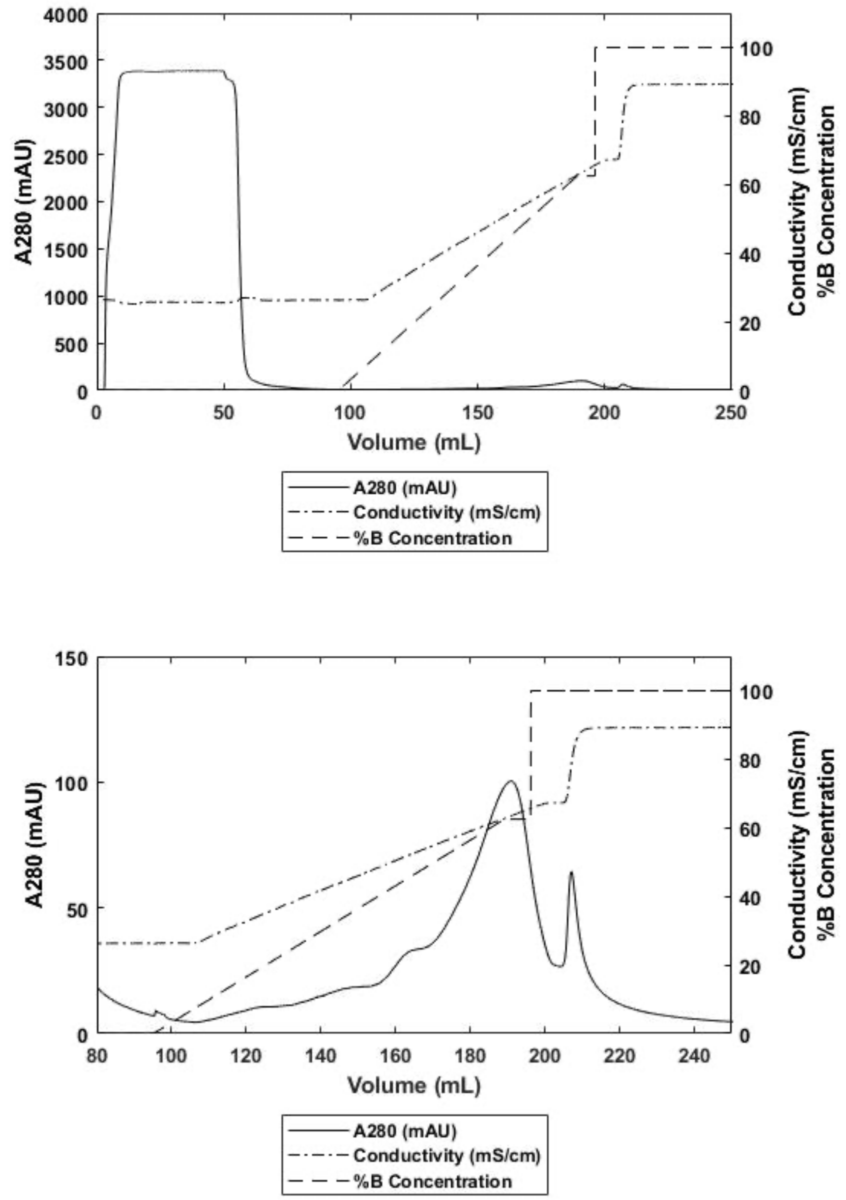

Figure S1. Overview chromatogram of PCV2 purification (top), and washing step with salt gradient chromatogram (bottom) where NaCl was varied from 200 to 700 mM in Tris-HCl buffer.

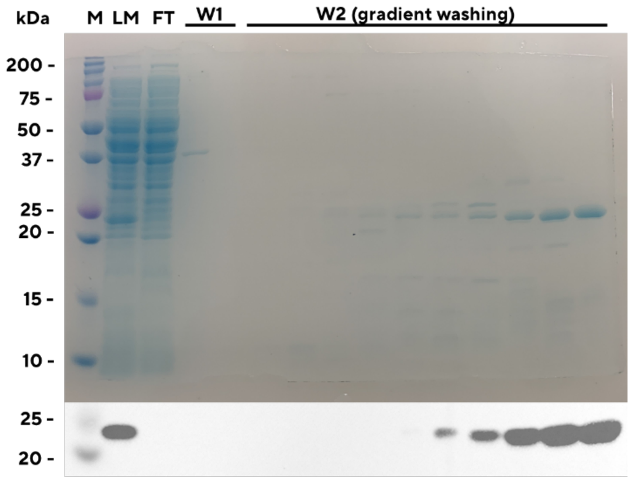

Figure S2. SDS-PAGE and western blot results of gradient washing step using salt concentration 200 to 700 mM NaCl in Tris-HCl buffer.

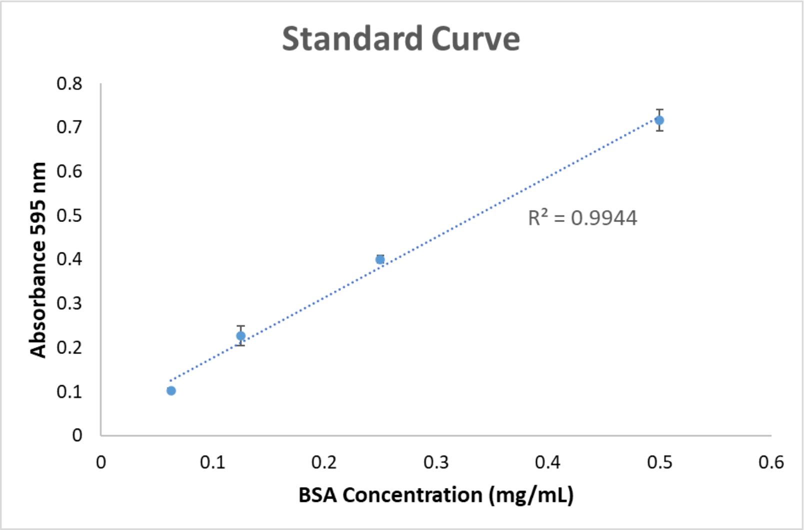

Protein Concentration

Fraction	Concentration (mg/mL)
E1	2.09
E2	7.11
E3	2.75
E4	1.36
E5	1.21
E6	0.85
E7	0.65
E8	1.00
E9	1.00
E10	0.98
E11	0.96
E12	0.82
E13	1.02

Figure S3. Standard curve (left) and protein concentration (right) of elution fractions from HiScale SP Sepharose Fast Flow.
